# Superior mesenteric vein absence with intestinal malrotation: a case report

**DOI:** 10.1186/s12893-022-01490-6

**Published:** 2022-02-03

**Authors:** Peter Dubovan, Miroslav Tomáš, Jana Pavlendová, Jozef Dolník, Ramadan Aziri, Daniel Pinďák

**Affiliations:** 1 Department of Surgical Oncology, National Cancer Institute Bratislava, Klenova 1, 833 10 Bratislava, Slovak Republic; 2 Department of Surgical Oncology, Faculty of Medicine, Slovak Medical University, Klenova 1, 833 10 Bratislava, Slovak Republic

**Keywords:** Mesenteric vein, Absence, Intestinal malrotation, Anaemia, Case reports

## Abstract

**Background:**

Congenital abnormalities are not very common and are even rarer when two or more are combined. Congenital malformation of the superior mesenteric vein may not affect normal development, or it may lead to moderate or even severe symptoms. In combination with intestinal malrotation, however, it may lead to the need for surgical intervention in the early years of life.

**Case presentation:**

We present the case of a 22-year-old patient who had been diagnosed with iron deficiency anaemia at the age of two months. As a result of the absence of the proximal section of the superior mesenteric vein, the patient has always needed iron supplements and an occasional erythrocyte transfusion. This has resulted from the formation of collaterals throughout the small bowel, causing chronic blood loss with its clinical manifestation. Although, there are some congenital abnormalities of the superior mesenteric vein, the absence of the superior mesenteric vein is rare, and in this case the clinical course was quite severe. Therefore, we planned bypass surgery for this patient to reduce the duodenal collaterals and resolve the persistent anaemia caused by chronic blood loss from the duodenum. We successfully performed the surgery consisting of the formation of anastomosis between the large collateral vein from the distal end of the superior mesenteric vein and the anterior inferior pancreaticoduodenal vein.

**Conclusion:**

The purpose of this case report is to describe the rare anatomical malformation of the superior mesenteric vein accompanied by intestinal malrotation, with its potential clinical implications regarding symptoms, clinical presentation, and the impact on potential surgery planning.

## Background

The healthy development of a human being may be affected by a variety of genetic and environmental factors resulting in congenital abnormalities which are either asymptomatic or lead to symptom presentation necessitating medical intervention. Independently, intestinal malrotation is not unique, with an incidence of 1:500 to 1:5000 live births [1]. In severe cases, it can lead to midgut volvulus and potentially result in intestinal ischemia. However, it may also cause only mild symptoms and thus often be a silent constituent in combination with other congenital malformations. The absence of the superior mesenteric vein (SMV) is rare and only a number of papers describe such a congenital abnormality. The combination of these anomalies is unique and this report describes such a clinical case with its clinical presentation and subsequent medical intervention. This case report has been reported in line with the SCARE criteria [2].

## Case presentation

**Fig. 1:**
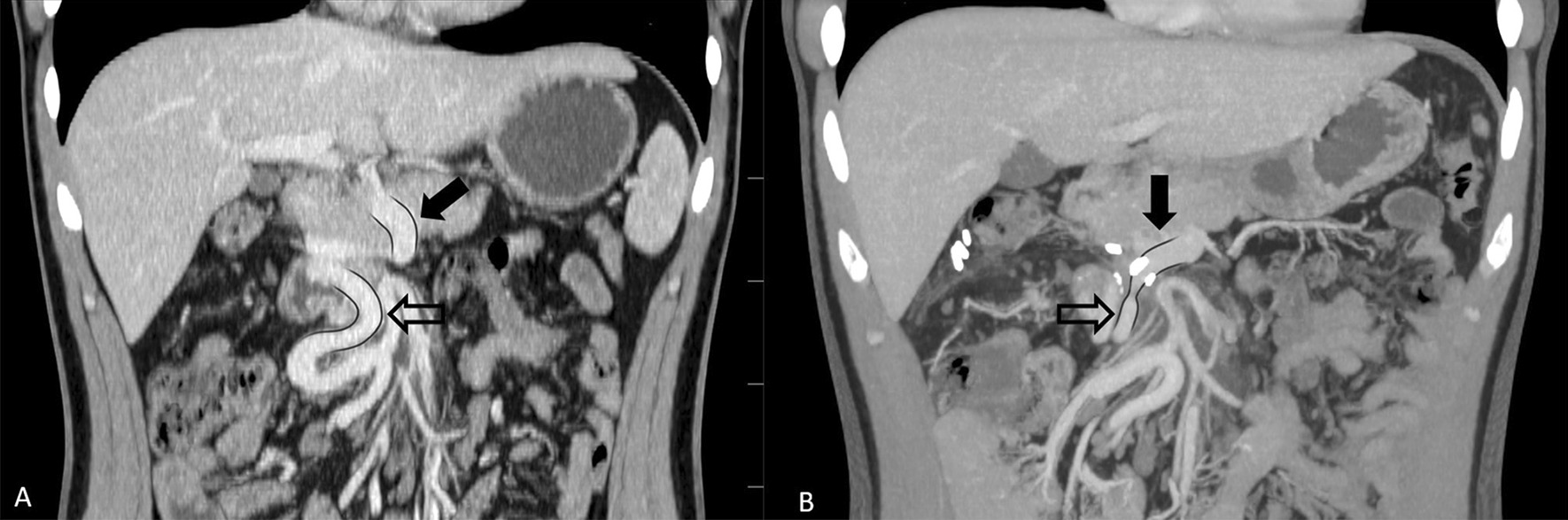
Abdominal CT—coronal sections. **A** Preoperative CT: the full black arrow points to the proximal section of the SMV, the contoured black arrow points to a large collateral arising from the distal section of the SMV. **B** Postoperative CT shows patent anastomosis between collateral branch from the distal section of the SMV and the anterior inferior pancreaticoduodenal vein

**Fig. 2:**
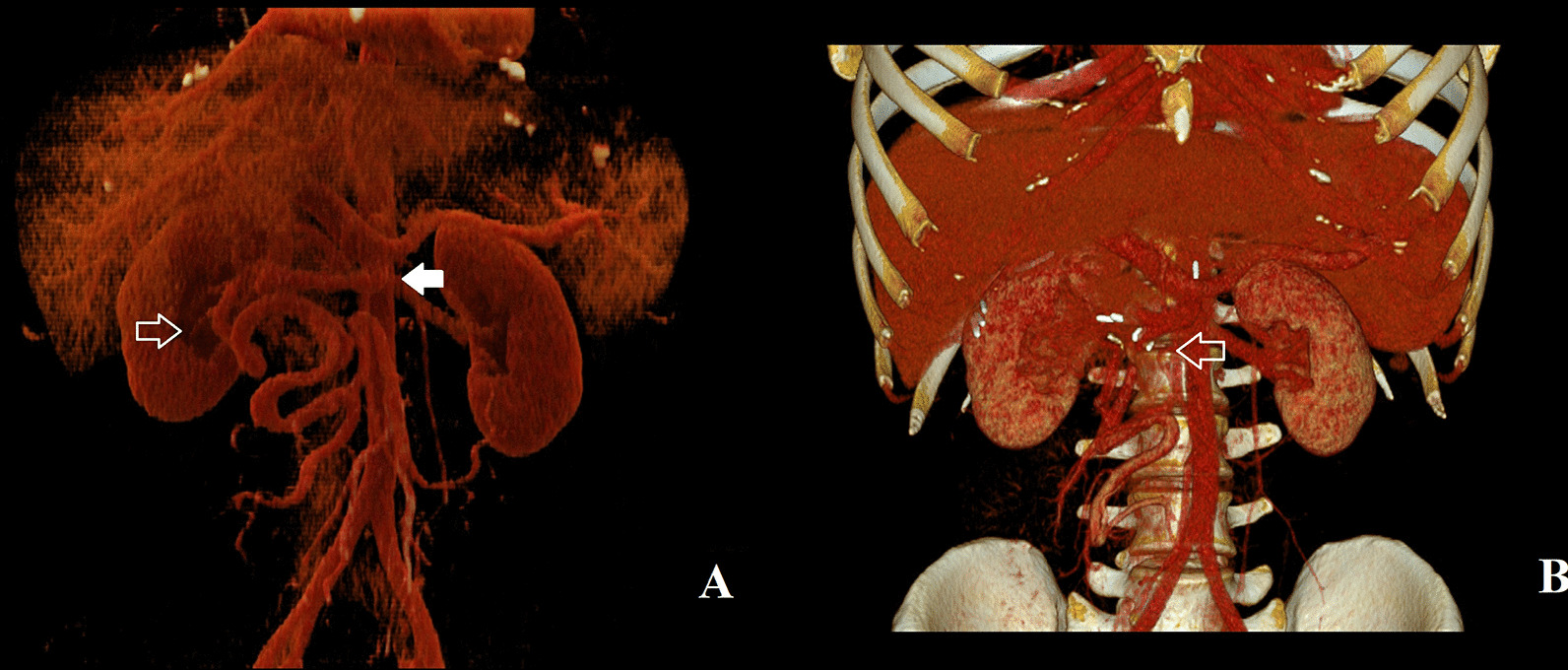
A 3D reconstructions of vascular system from coronal CT scans: **A** Preoperative CT reconstruction: the full white arrow points to the proximal section of the SMV, the contoured white arrow points towards collaterals running through the duodenum. **B** Postoperative CT reconstruction shows patent anastomosis between collateral branch from the distal section of the SMV and the anterior inferior pancreaticoduodenal vein

A 22-year-old Caucasian male was admitted to hospital with aggravated signs of anaemia: primarily exhaustion and sleepiness, accompanied by marked pallor. Iron deficiency anaemia had been diagnosed in this patient at the age of 2 months. The patient was then put on iron supplements along with other vitamins necessary for erythropoiesis. Before his first birthday, celiac disease was histologically diagnosed, and anaemia was considered to be an accompanying disease. In early childhood, the patient experienced a cytomegalovirus infection as well as other typical childhood diseases. He was immunized according to his home country’s specific vaccination schedule and had an otherwise unremarkable childhood. Even though the patient had been put on oral iron and vitamin supplements, the severity of the anaemia remained within the moderate to severe range, only sporadically mild, and therefore the patient underwent further testing. At the age of 18, the patient underwent bone marrow examination with no pathologic findings, as well as an upper gastrointestinal endoscopy, lower gastrointestinal endoscopy, and capsule endoscopy, all of which had done previously. This time, however, the discovery of dilated blood vessels in the duodenum was made during upper gastrointestinal endoscopy, although neither angiodysplasia nor Osler-Weber-Rendu disease were found. A computed tomography (CT) of the abdomen was performed resulting in the discovery of an intestinal malrotation of the duodenum and an absence of the proximal part of the SMV with varices in the duodenal wall, which had functioned as a collateral blood flow due to mesenteric vein occlusion (Figs. 1A, 2A). The anaemia was proclaimed to be a result of chronic occult blood loss through dilated blood vessels in duodenal mucosa, and therapy slightly helped the condition at the time.

**Fig. 3:**
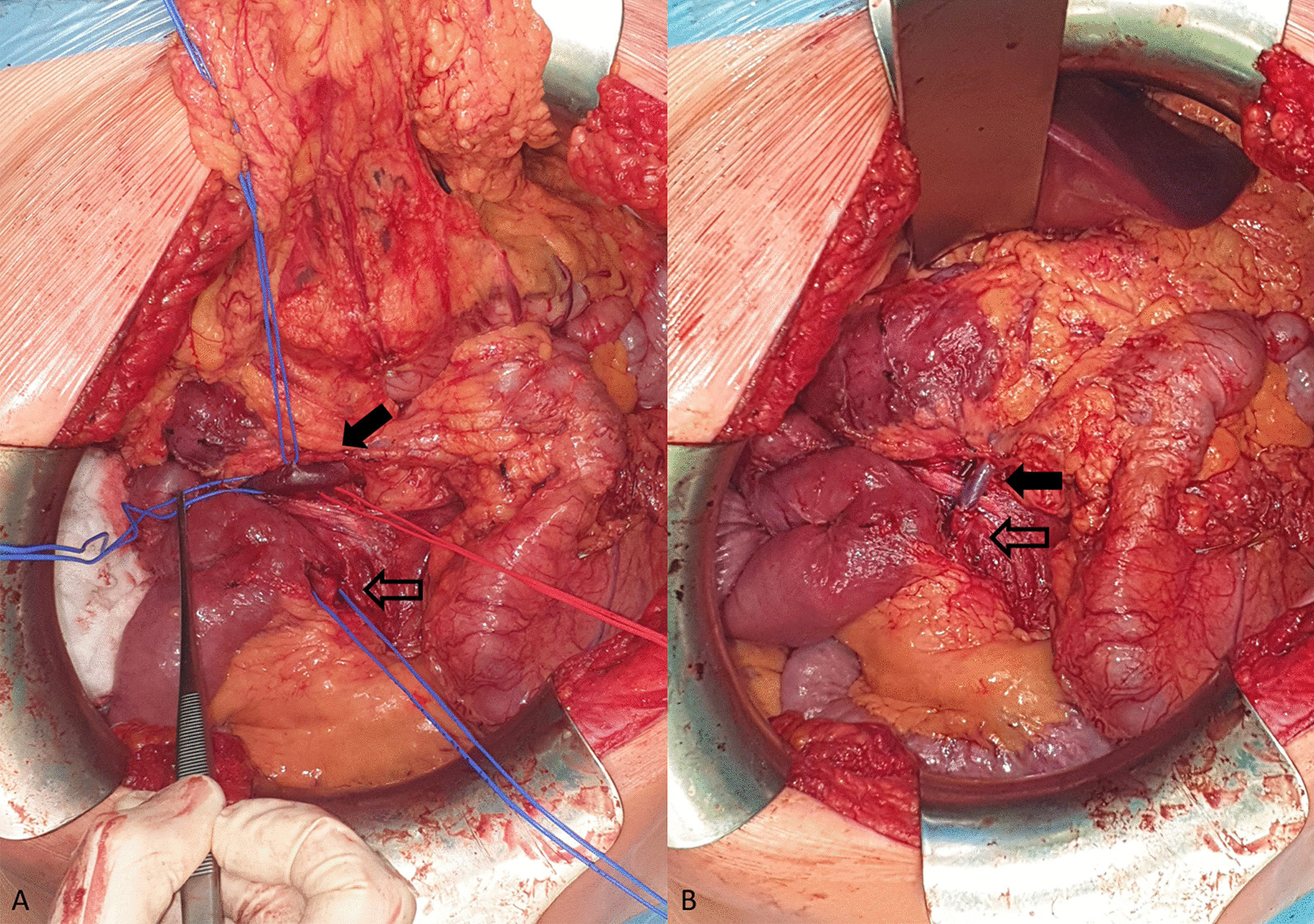
Intraoperative images before and after the reconstruction. **A** Before the reconstruction: the black arrow points to the anterior inferior pancreaticoduodenal vein, the contoured black arrow points to the large collateral arising from the distal section of the SMV. **B** After the reconstruction: image of the anastomosis between the collateral from the distal section of the SMV to the anterior inferior pancreaticoduodenal vein

At the age of 22, the patient quit taking all supplements. The resulting life-threatening anaemia necessitated hospitalization and red blood cell transfusions within 6 months. Although, the transfusions did not yield the expected results, they indirectly confirmed a chronic loss via the duodenal collaterals. The patient underwent another computed tomography of the abdomen which confirmed the previous findings, and, after his condition was stabilized, was referred to our surgical clinic for a consultation. An elective surgery was scheduled for the following month. Meanwhile, the patient was treated with repeated intravenous iron supplementation and correction of hemostasis parameters. Finally, when the patient was admitted to our surgical ward, he was slightly pale but a physical examination produced no remarkable findings. The next day, he underwent surgery under general anaesthesia with mobilisation of the malrotated bowel, and after meticulous preparation we were able to find the suitable blood vessels for the bypass. After administering an intravenous heparin (100 units per kg), we created an anastomosis between the large collateral arising from the distal section of the SMV and the anterior inferior pancreaticoduodenal vein draining to the present proximal section of the SMV without any prosthetic material (Fig. 3A, B). We also performed ligation of some dilated veins on the duodenal surface with the aim to interrupt the blood flow in the duodenal varices. The postoperative period was unremarkable, and the patient was discharged to outpatient care. During the 3-month period after the surgery, we put the patient on anticoagulant treatment with low molecular weight heparins. Five months after the surgical intervention, a CT of the abdomen (Figs. 1B, 2B) was performed to confirm the patency of the venous anastomosis and diminution of duodenal varices. A slightly stenotic passage through the anastomosis with a regression of the duodenal varices, draining into the SMV, was observed. Two months later, the patient underwent an upper gastrointestinal endoscopy with the intention to ligate the residual duodenal varices. The patient still has three large duodenal varices, which have been currently left untreated because of the patient’s good clinical and laboratory status with no clinical signs of bleeding and no anaemia or iron deficiency.

## Discussion

Human embryogenesis is a very delicate process during which the development of the SMV and rotation of the intestine starts approximately at the same time [1, 3]. The superior mesenteric vein develops secondarily to the regression of the left vitelline vein in the midgut mesentery [3]. After formation it runs slightly anterior and to the right of the superior mesenteric artery most of the time. However, this course may vary in different ways and occasionally the main trunk of the SMV is not formed and the jejunal branches merge at the level of the splenic vein [4]. A different course of the SMV may be a result of an intestinal malrotation as well and therefore, it seems, that the two processes may be intertwined on a molecular level.

Changes in the anatomy of the portal venous system may arise from different causes as well. Such source may be the congenital absence of the portal vein (CAPV). The development of the portal vein (PV) occurs approximately in the same fetal period as the development of the SMV [3, 5]. The vitelline veins play important role in both the processes, although changes in the development of portal vein seem to be determined by the extent of the insult, resulting either in complete absence of the PV or the portosystemic shunt. Congenital portosystemic shunt is much more frequent compared to the literature mentions of the SMV absence with the estimated incidence to be 1:30,000 people [5]. The formation of the SMV and PV have some things in common, however based on the CAPV frequency and determined findings such as an association with the congenital heart disease or genetic syndromes (Down’s and Turner’s syndrome), CAPV and the absence of the SMV have probably different etiology [5, 6].

Only a few scientific papers recognised the absence of the SMV, but it was only secondary to another problem (Table 1). In one case study from 1964, Blough et al. [7] describes the discovery of the absence of the SMV when the patient was admitted for recurrent intestinal malrotation with a necessity for intestinal resection. It was only then they discovered the problem with the venous intestinal outflow regarded as a result of the absence of the SMV with the collateral running through the mesocolon. Similarly, Tanaka et al. 2019 [8] faced a challenging situation when performing Whipple procedure in a 74-year-old patient eventually diagnosed with pancreatic intraductal papillary mucinous neoplasm with the absence of superior mesenteric vein.

**Table 1:** Published cases of SMV absence

Blough et al. (1964) [7]	SMV absence with small bowel draining through the mesocolon into left colic vein	12 year old patient	Male	Surgical diagnosis: recurrent intestinal volvulus	Emergency surgery—bowel resection with the need for a vascular reconstruction	Asymptomatic course of the SMV absence—accidental finding
Tanaka et al. (2019)[8]	SMV absence with small bowel and right colon draining into inferior mesenteric vein	74 year old patient	Female	Surgical diagnosis: Intraductal papillary mucinous neoplasia of pancreatic head	Elective surgery—cephalic duodenopancreatectomy	Asymptomatic course of the SMV absence—accidental finding

In our case study, a progressive worsening of the patient’s clinical condition was observed and therefore, it was presumed, the reason behind it is the absence of the proximal part of the SMV, as was discovered on the CT scans. The tortuous course of the duodenal collaterals along with duodenal submucous varices caused chronic occult blood loss with its clinical manifestation and it posed the major risk of a bleeding event with the possibility of serious adverse effects. Therefore, the surgery was planned with an attempt to create a bypass without the use of prosthetic material in order to keep clear of the lifelong necessity for anticoagulant therapy, as the patient at the time of surgery was only 22 years old. In our case study the intestinal malrotation was only an accompanying developmental abnormality, which had silent or no clinical presentation.

To conclude, even though the literature mentions of the absence of the SMV are scarce, it may be an unrecognised congenital abnormality accompanying intestinal malrotation resulting in potentially serious clinical manifestation. The aim of this case study was to bring attention to such a clinical scenario as well as to draw attention to pancreatic surgery planning, as this condition may also have only mild or no clinical presentation and result in more complicated surgery.

## Data Availability

The authors can confirm that all relevant data are included in the article.

## Abbreviations

CAPV: Congenital absence of the portal vein
CT: Computed tomography
PV: Portal vein
SMV: Superior mesenteric vein
SCARE: Surgical case report

## References

[1] Škába R. Střevní malrotace. In: Pechan J, Haruštiak S, Kothaj P, Vajó J, Siman J, editors. Princípy chirurgie III. 2. Edition. Bratislava. Slovak Academic Press. 2015. p. 923–927. ISBN 978–80–89607–32–7.

[2] Agha RA, Borrelli MR, Farwana R (2018). The SCARE 2018 statement: updating consensus surgical CAse REport (SCARE) guidelines. Int J Surg.

[3] Abe H, Yamamoto M, Yanagisawa N (2017). Regressing vitelline vein and the initial development of the superior mesenteric vein in human embryos. Okajimas Folia Anat Jpn.

[4] Katz MHG, Fleming JB, Pisters PWT (2008). Anatomy of the superior mesenteric vein with special reference to the surgical management of first-order branch involvement at pancreaticoduodenectomy. Ann Surg.

[5] Sanada Y, Mizuta K (2018). Congenital absence of the portal vein: translated version. J Hepato-Billiary-Pancreat Sci.

[6] Hu GH, Shen LG, Yang J (2008). Insight into congenital absence of the portal vein: is it rare?. World J Gastroenterol.

[7] Blough JB, Smith PD (1964). Malrotation of midgut associated with absence of superior mesenteric vein outflow. Am J Surg.

[8] Tanaka K, Miyamoto A, Asaoka T (2019). Pancreatic intraductal papillary mucinous neoplasm with anomaly of the portal venous system: absence of superior mesenteric vein. Jpn J Gastroenterol Surg..

